# PARP inhibition in UV-associated angiosarcoma preclinical models

**DOI:** 10.1007/s00432-021-03678-4

**Published:** 2021-06-03

**Authors:** Marije E. Weidema, Ingrid M. E. Desar, Melissa H. S. Hillebrandt-Roeffen, Anke E. M. van Erp, Mikio Masuzawa, PALGA-group, J. W. R. Meyer, M. C. H. Hogenes, Uta E. Flucke, Winette T. A. van der Graaf, Yvonne M. H. Versleijen-Jonkers

**Affiliations:** 1 Department of Medical Oncology, Radboud University Medical Center, Internal Postal Code 452, P.O. Box 9101, 6500 HB Nijmegen, The Netherlands; 2 Department of Regulation Biochemistry, Kitasato University School of Allied Sciences, Sagamihara, Japan; 3 Department of Pathology, Radboud University Medical Center, Nijmegen, The Netherlands; 4 Department of Medical Oncology, Netherlands Cancer Institute, Amsterdam, The Netherlands

**Keywords:** Angiosarcoma, Subtype, PARP, Temozolomide, Combination treatment, SLFN11

## Abstract

**Purpose:**

Angiosarcoma (AS) is a rare vasoformative sarcoma, with poor overall survival and a high need for novel treatment options. Clinically, AS consists of different subtypes, including AS related to previous UV exposure (UV AS) which could indicate susceptibility to DNA damage repair inhibition. We, therefore, investigated the presence of biomarkers PARP1 (poly(ADP-ribose)polymerase-1) and Schlafen-11 (SLFN11) in UV AS. Based on experiences in other sarcomas, we examined (combination) treatment of PARP inhibitor (PARPi) olaparib and temozolomide (TMZ) in UV AS cell lines.

**Methods:**

Previously collected UV AS (*n* = 47) and non-UV AS (*n* = 96) patient samples and two UV AS cell lines (MO-LAS and AS-M) were immunohistochemically assessed for PARP1 and SLFN11 expression. Both cell lines were treated with single agents PARPi olaparib and TMZ, and the combination treatment. Next, cell viability and treatment synergy were analyzed. In addition, effects on apoptosis and DNA damage were examined.

**Results:**

In 46/47 UV AS samples (98%), PARP1 expression was present. SLFN11 was expressed in 80% (37/46) of cases. Olaparib and TMZ combination treatment was synergistic in both cell lines, with significantly increased apoptosis compared to single agent treatment. Furthermore, a significant increase in DNA damage marker γH2AX was present in both cell lines after combination therapy.

**Conclusion:**

We showed combination treatment of olaparib with TMZ was synergistic in UV AS cell lines. Expression of PARP1 and SLFN11 was present in the majority of UV AS tumor samples. Together, these results suggest combination treatment of olaparib and TMZ is a potential novel AS subtype-specific treatment option for UV AS patients.

**Supplementary Information:**

The online version contains supplementary material available at 10.1007/s00432-021-03678-4.

## Introduction

Angiosarcoma (AS) is a rare sarcoma with endothelial properties and an incidence of 0.15 per 100.000 persons per year (NCIN 2012). AS can occur either sporadically (primary AS) or due to external damaging factors such as UV exposure, radiation therapy or chronic lymphedema (secondary AS). Localized AS treatment consists of surgery, combined with (neo-)adjuvant radiation therapy or chemotherapy. Patients with metastatic disease usually receive chemotherapy, either anthracycline-based regimens or paclitaxel (Penel et al. 2012). Later lines of treatment can include other chemotherapeutic agents such as gemcitabine (Stacchiotti et al. 2012) or targeted therapy with pazopanib (van der Graaf et al. 2012; Kollar et al. 2017). Reported overall survival (OS) of all AS patients is poor with a 5-year survival rate of around 22–40% (Lahat et al. 2010; Wang et al. 2017; Weidema et al. 2019), dropping to only 15% for metastatic patients (Lahat et al. 2010). To improve AS prognosis, several targeted treatment strategies have been explored, such as bevacizumab or sorafenib (Agulnik et al. 2013; Ray-Coquard et al. 2012). Recently, small case series have suggested a role for immunotherapy in selected AS patients (Painter et al. 2020; Florou et al. 2019). Although these results are promising, these efforts have yet to result in clinical implications for current AS patients.

Over the past decades, AS patient survival has not improved, emphasizing the need for more effective treatment options (Zhang et al. 2019). As a start, more in-depth knowledge on the molecular characteristics of the different AS subtypes would be instrumental for further research (Chan et al. 2020; Painter et al. 2020; Weidema et al. 2020). A recent study provided evidence for the role of UV-induced DNA damage in a subset of AS, showing that 10/12 (83%) cutaneous AS originating in the head and neck region harbored a genetic UV signature (Painter et al. 2020). The fact that UV-associated AS develop due to DNA damage, gave rise to the hypothesis that these tumors could be particularly susceptible to inhibition of the DNA damage response (DDR) pathway.

The DDR machinery includes base excision repair (BER); a major pathway involved in among others repair of DNA single strand breaks (SSBs) generated by reactive oxygen species, ionizing radiation and alkylating agents (Zhang et al. 2012). A key protein in the BER pathway is poly(ADP-ribose) polymerase-1 (PARP1), which initiates repair by detection of SSBs (Lord and Ashworth 2012). Upon binding of PARP1 to the SSB, PARP1 initiates formation of poly ADP-ribose (pADPr) chains. Both PARP1 and the pADPr chains then attract proteins to form the BER complex. Next, release of PARP1 from the damaged DNA allows access for BER proteins to the damaged site, thus enabling repair. Upon PARP inhibition, SSB repair is compromised leading to persistent SSBs. In addition, release of PARP1 from the DNA is inhibited (so-called PARP trapping). Both persistent SSBs as well as PARP trapping can stall replication forks, potentially resulting in lethal double-strand breaks (DSBs).

In case of defects in the repair of DSBs, for instance in BRCA-mutated cancers, PARP inhibitor (PARPi) monotherapy can already effectively cause cell death (Farmer et al. 2005). However, in malignancies without such defects, clinical application of PARPi monotherapy was shown to have only limited efficacy (Choy et al. 2014; Leichman et al. 2016). PARP inhibitors have been shown to synergize with different kinds of chemotherapeutic agents. Of these drugs, TMZ induces SSBs and causes increased PARP trapping when combined with a PARPi (Murai et al. 2014), and combination therapy of PARPi with TMZ has shown promising results in vitro and in vivo in other sarcomas, such as Ewing sarcoma, desmoplastic small round cell tumors and chordoma (Smith et al. 2015; Cao et al. 2019; van Erp et al. 2020). We hypothesized that combination treatment of a PARPi with TMZ would be of particular interest in UV AS, based on the UV-induced DNA damage already present in the cells, as well as additional DNA damage inflicted by TMZ.

To assess the potential of this combination in UV AS, we aimed to study the presence of biomarkers for response. PARP1 expression was shown to be predictive of response to combination treatment of PARPi olaparib with trabectedin and other cytotoxic agents across different cell lines (Pignochino et al. 2017). In addition, PARP1 expression was shown to be crucial for response to PARPi, as PARP1 knockdown made cells highly resistant to PARP inhibition (Murai et al. 2012). Schlafen-11 (SLFN11) is an even more interesting potential biomarker. It is recruited to stressed replication forks, then opens chromatin and thus induces a permanent and lethal replication block in cells under replication stress (Murai et al. 2018). Based on this mechanism, SLFN11 is a biomarker for response to agents causing replicative stress, including PARP inhibitors (Zoppoli et al. 2012; Barretina et al. 2012; Murai et al. 2016). Knockdown of SLFN11 has shown to result in decreased sensitivity to the DNA damaging cytotoxic agent trabectedin in preclinical liposarcoma and Ewing sarcoma (ES) models (Iwasaki et al. 2019) and decreased sensitivity to the PARPi talazoparib in small cell lung cancer (SCLC) cell lines (Lok et al. 2017). As for the combination therapy of PARPi and TMZ, SLFN11 expression appears to mainly correlate with sensitivity to PARPi treatment, however, in mesothelioma cell lines and SCLC in vivo models SLFN11 expression also correlated with increased response to PARPi and TMZ combination treatment (Lok et al. 2017; Rathkey et al. 2020). Furthermore, resistance to the combination of TMZ with PARPi niraparib was shown after SLFN11 knockdown in ES cells (Tang et al. 2015).

We, therefore, aimed to assess the presence of PARP1 and SLFN11 protein expression in clinically derived tumor tissue of UV AS and non-UV AS cases. Next, we investigated whether combination therapy of TMZ and PARPi olaparib is effective in preclinical in vitro UV AS models.

## Materials and methods

### Immunohistochemical PARP1 and SLFN11 expression in patient-derived AS tumor tissue

In a previous study, AS cases and clinical data were collected via a nationwide query in The Netherlands (1989–2014) using the Netherlands Cancer Registry (NCR) (Weidema et al. 2019). Through linkage with the Dutch Nationwide Network and Registry of Histo- and Cytopathology (PALGA), AS tumor samples were collected for pathology review (Casparie et al. 2007). Confirmed AS cases were categorized based on clinical origin, with all cutaneous AS from the sun-exposed skin of the head and neck region classified as UV-associated. Non-UV AS cases were classified according to clinical subtype; including radiotherapy-induced, cutaneous non-UV associated, Stewart Treves (associated with chronic lymphedema) and visceral angiosarcoma. From tissue microarrays (TMAs), constructed from formalin-fixed, paraffin-embedded (FFPE) material of AS cases of which sufficient tumor was available with one or two 2.0 mM cores per tumor sample from representative tumor areas, 4 µM thick slides were assessed for PARP1 and SLFN11 expression. For PARP1, colon and tonsil tissue served as positive control. For SLFN11, tonsil and a xenograft of a human Ewing sarcoma served as positive control. Sections were deparaffinized in xylene and rehydrated through a graded ethanol into water series. Antigen retrieval was performed by heating the slides in EDTA buffer, pH9 for 10 min (PARP1) or 20 min (SLFN11) at 100 °C. Endogenous peroxidase activity was blocked with 3% H_2_O_2_ in distilled water for 10 min at room temperature. Subsequently, sections were incubated with monoclonal rabbit anti-PARP1 antibody (1:800, clone E102, Abcam, Cambridge, UK) or monoclonal rabbit anti-SLFN11 antibody (1:100, clone D8W1B, Cell Signaling Technology, Leiden, The Netherlands) in antibody diluent in a humidified chamber overnight at 4 °C. Next, tissue sections were incubated with Poly-HRP-GAMs/Rb IgG (ImmunoLogic, Duiven, The Netherlands) in EnVision™ FLEX Wash Buffer (Dako, Agilent Technologies, Santa Clara, CA, USA) (1:1) for 30 min at room temperature. Antibody binding was visualized using the EnVision™ FLEX Substrate Working Solution (Dako) for 10 min at room temperature. Finally, slides were counterstained with haematoxylin, dehydrated and coverslipped.

After validation, all IHC stainings were scored by two independent observers. For PARP1 and SLFN11, cores with > 50% tumor cells with positive nuclear staining were regarded positive. In case of a discrepancy between two cores derived from one patient, the highest score was applied, whereas in case of observer discrepancies, a third observer was consulted. Correlation analysis to assess the correlation between SLFN11 and PARP1 expression was performed using the Fisher’s exact test, with IBM SPSS Statistics (Armonk, NY, USA), version 25.0.0.1. This study was performed in accordance with the Code of Conduct of the Federation of Medical Scientific Societies in The Netherlands.

### Cell lines, cell culture and compounds

Human cutaneous scalp AS cell line MO-LAS was generously provided by Dr. Masuzawa from the Kitasato University School of Allied Sciences, Sagamihara, Japan (Masuzawa et al. 2012). MO-LAS cells were cultured in Dulbecco’s Modified Eagle’s Medium (DMEM, Lonza, The Netherlands) supplemented with 10% Fetal Bovine Serum (FBS, Gibco, Thermo Fisher Scientific, Waltham, MA, USA) and 1% penicillin–streptomycin (Gibco). Human cutaneous scalp AS cell line AS-M was generously provided by Prof. Kirkpatrick and Dr. Unger from Johannes Gutenberg University, Mainz, Germany (Krump-Konvalinkova et al. 2003). AS-M cells were cultured in Endothelium Cell Growth Medium MV (ECGM MV, PromoCell, Heidelberg, Germany) and 1% penicillin–streptomycin. Similar to the tumor samples, both cell lines originated from sun-exposed skin of the scalp. Both MO-LAS and AS-M cells were kept in a humidified atmosphere of 5% CO_2_/95% air at 37 °C. TMZ and olaparib were purchased from Selleckchem (Houston, TX, USA) and diluted in DMSO for in vitro experiments. Experiments were performed in triplicate.

### Cell viability assay

Cell viability was assessed using the MTT assay. Cells were seeded in quadruplicate in a 96-wells plate (3000 cells/well) and allowed to adhere overnight. Cells were then treated in a range of concentrations of TMZ (0–500 µM) or olaparib (0–100 µM) monotherapy for 144 h. Next, cells were incubated with 5 mg/ml MTT (Sigma-Aldrich, Saint Louis, MO, USA) in PBS for 3.5 h. The formed formazan crystals were dissolved in MTT solvent (isopropanol, 0.1% NP-40 and 4 mM fuming hydrochloric acid). Absorbance was measured with a dual measurement at 560 and 655 nm using the Bio-Rad iMark plate reader (Bio-Rad, Hercules, CA, USA). For each drug the IC50 was calculated using GraphPad Prism software (version 5.03 for Windows, GraphPad Software, San Diego, CA, USA, http://www.graphpad.com).

### Drug synergy and combination index

Drug synergy of combined TMZ and olaparib was assessed by calculation of the combination index (CI) and dose reduction index (DRI) with CompuSyn software (ComboSyn Inc.) using the Chou-Talalay method (Chou 2006). Cells were simultaneously treated with TMZ and olaparib concentrations in a non-constant ratio for 144 h, combining relatively low dosages of olaparib (using the IC_50_ and approximately 50% of the IC_50_ values) with increasing dosages of TMZ (10; 25; 50; 100 µM). Effects on cell viability of the monotherapy and combination therapy were assessed in three independent experiments, using an average fraction of cell viability affected (FA) value for further calculations. Results of the combination treatment are represented in an isobologram, in which the line represents an additive effect (CI = 1.0) of the combination at the given FA value. Points below the line represent synergism (CI < 1.0), points above the line represent antagonism (CI > 1.0) (Chou 2006). More specifically, according to Chou et al., a CI value between 0.3 and 0.7 indicates synergism and CI 0.1–0.3 strong synergism (Chou 2006). The *X*- and *Y*-axis represent the fraction of the dose necessary as a single agent to generate reduction of *x*% cell viability (*D*_1/2_) divided by the portion of the drug in the combination treatment (*D*_1_ + *D*_2_) necessary to reduce a similar *x*% cell viability (*Dx*)_1/2_. DRI values of > 1.0 indicate a favorable dose reduction in the combination treatment compared to the monotherapy dose. Differences in cell viability following combination treatment were analyzed by two-way ANOVA with Bonferroni post-test using GraphPad Prism software, a *p* value < 0.05 was considered significant (*< 0.05, **< 0.01, ***< 0.001).

### Western blot

Western blot analysis of γH2AX was performed to investigate the induction of DNA damage after single agent and combination treatments with low and high dose TMZ. Protein extracts were purified from the MO-LAS and AS-M cell lines after 48 h with single agent and combination treatment. Cells were incubated in cold RIPA buffer containing protease and phosphatase inhibitors and the lysates were centrifuged at 14,000*g* at 4 °C for 15 min. Then protein concentrations of the supernatants were determined with the BCA protein assay system (Pierce Endogen, Rockford, IL, USA). Of each condition, equal amounts of protein (50 μg) were loaded and run on a 12% sodium dodecyl sulfate–polyacrylamide gel electrophoresis (SDS-PAGE) gel under reducing conditions and subsequently transferred to nitrocellulose membranes. After blocking with Odyssey blockbuffer (LI-COR Biosciences, Lincoln, NE, USA) in TBS (1:1) at room temperature (RT) for 1 h, membranes were incubated overnight at 4 °C with monoclonal rabbit anti-phospho-Histone H2A.X (γH2AX, Ser139) (1:1000, Cell Signaling Technology, Leiden, The Netherlands). Next, the blots were incubated at room temperature for 1 h with a goat-anti-rabbit fluor 680-conjugated secondary antibody (1:5000, AlexaFluor, Invitrogen, OR, USA), incubated for 1 h at RT with monoclonal mouse anti-GAPDH (1:10,000, Abcam, Cambridge, UK) as a loading control and subsequently incubated for 1 h at RT with a goat-anti-mouse fluor 800-conjugated secondary antibody (1:5000, AlexaFluor, Invitrogen). The fluorescent signals were analyzed with the Odyssey Infrared Imaging System (LI-COR Biosciences) and Odyssey Application Software (version 3.0.30). The experiment was performed in duplicate. In addition, western blot analysis of PARP1 and SLFN11 was performed as described above on the MO-LAS and AS-M cell line, using the monoclonal rabbit anti-PARP1 (#9542, 1:2000, Cell Signaling Technology), monoclonal rabbit anti-SLFN11 (#34858, 1:1000, Cell Signaling Technology), and monoclonal mouse anti alpha Tubulin (A11126, 1:1000, Invitrogen).

### Apoptosis assay

Apoptosis was assessed using the Annexin-V/propidium iodide (PI) double staining apoptosis assay (Biovision Cat# 1001-200, CA, USA) after single agent treatment and combination treatment. Cells were incubated with Annexin-V-FITC and PI in cell culture medium supplemented with CaCl_2_ (final concentration 15 mM). Apoptotic cells were measured using the CytoFLEX flow cytometer (Beckman-Coulter, Brea, CA, USA) and the percentage of early (Annexin-V positive, PI negative) and late (Annexin-V and PI positive) apoptotic cells was calculated using FlowJo software (version 10.0.7).

## Results

### PARP1 and SLFN11 expression in AS tumor tissues

**Fig. 1:**
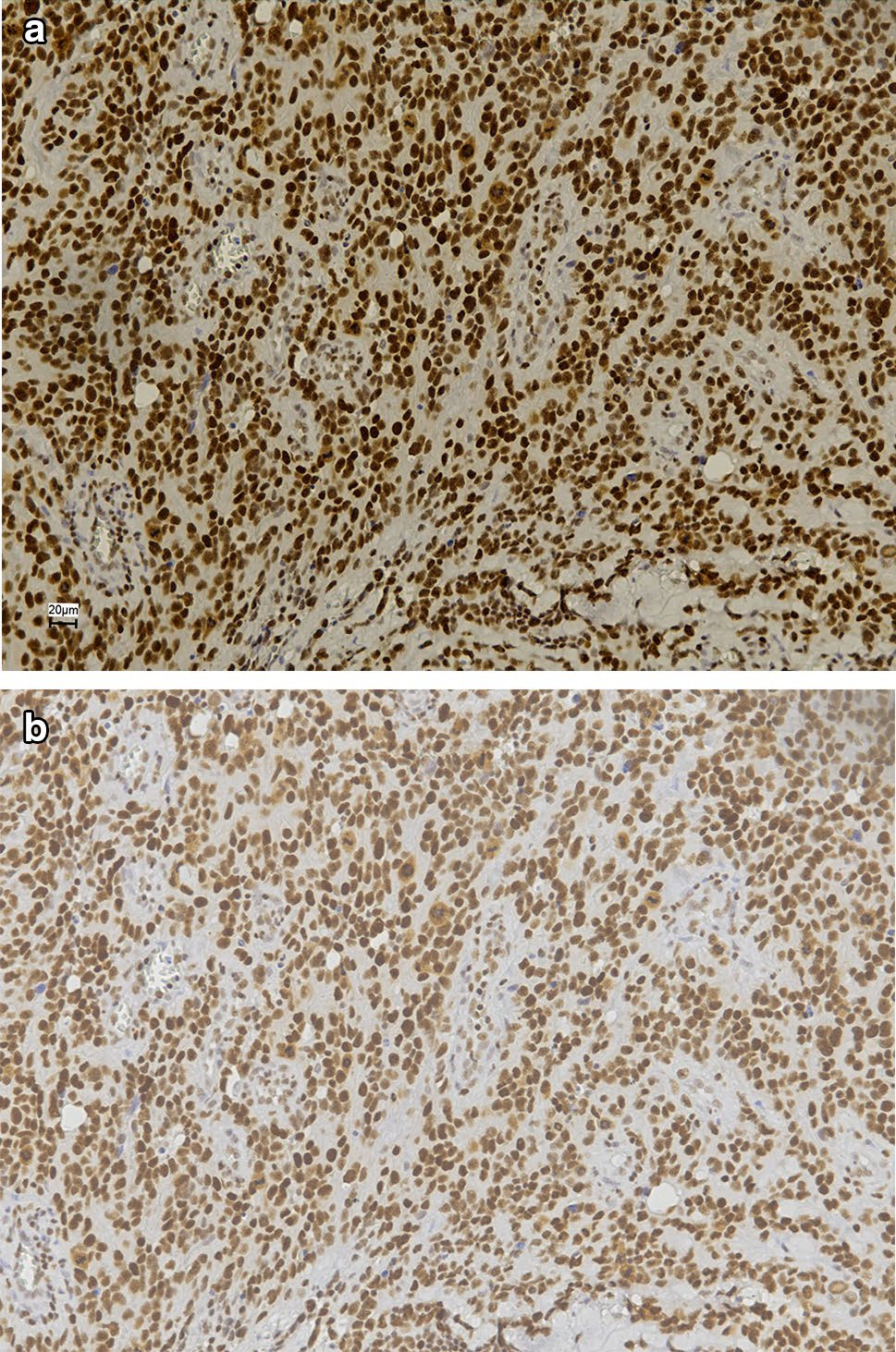
Immunohistochemical expression of PARP1 and SLFN11. **a** UV AS sample positive for PARP1. **b** UV AS sample showing SLFN11 expression. Images taken at × 20 digital magnification

A total of 47 UV AS patients were assessed for immunohistochemical expression of PARP1 and SLFN11. Median age was 78 years and median overall survival was 13 months (range 0–194 months). Additional clinical characteristics are described in Supplemental Table 1 PARP1 was expressed in 46/47 (98%) of cases, whereas 80% (37/46) of tumors showed SLFN11 positivity (Fig. 1, Table 1; Supplemental Fig. 1). In 36 UV AS patients (77%), both SLFN11 and PARP1 expression was present. None of the UV AS samples was negative for both PARP1 and SLFN11 expression. In addition, tumor samples of 96 non-UV AS patients were assessed for PARP1 and SLFN11 expression, showing 89–100% PARP1 positivity and 63–66% cases positive for SLFN11 expression. In all 143 AS cases taken together, SLFN11 and PARP11 expression was significantly correlated (*p* = 0.002, Supplemental Table 2).

**Table 1:** SLFN11 and PARP1 immunohistochemical expression per AS subtype

AS subtype	SLFN11+ (%*)	SLFN11− (%*)	SLFN11 Not evaluable	PARP1+ (%*)	PARP1− (%*)	PARP1 Not evaluable
UV AS (*n* = 47)	37 (80)	9 (20)	1	46/47 (98)	1/47 (2)	0
Non-UV AS (*n* = 96)	61 (66)	31 (34)	4	80 (93)	6 (7)	10
Cutaneous non-UV AS (*n* = 19)	12 (67)	6 (33)	1	17/17 (100)	0	2
RT AS (*n* = 32)	20 (67)	10 (33)	2	29/32 (91)	3/32 (9)	0
StwT AS (*n* = 16)	10 (63)	6 (37)	0	10/10 (100)	0	6
Visc AS (*n* = 29)	19 (68)	9 (32)	1	24/27 (89)	3/27 (11)	2
Total (*n* = 143)	98 (71)	40 (29)	5	126/133 (95)	7/133 (5)	10

*UV AS* UV-associated angiosarcoma, *RT AS* radiotherapy-induced angiosarcoma, *StwT AS* Stewart Treves angiosarcoma, *Visc AS* visceral angiosarcoma

*Of evaluable cases

Both the MO-LAS and the AS-M cell line showed strong immunohistochemical positivity for PARP1 and SLFN11, as well as presence of PARP1 and SLFN11 by western blot analysis (Supplemental Fig. 2).

### Combination treatment of olaparib and TMZ in AS cell lines

**Fig. 2:**
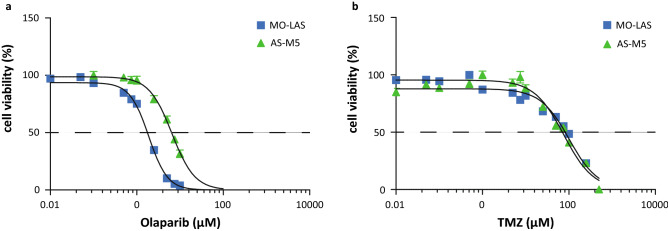
Single agent therapy effects on cell viability of olaparib and TMZ. **a** Cell viability after treatment with olaparib for MO-LAS and AS-M cells (mean values of three experiments); **b** Cell viability after treatment with temozolomide for MO-LAS and AS-M cells (mean values of three experiments). Dotted line represents 50% reduction in cell viability (IC_50_)

**Fig. 3:**
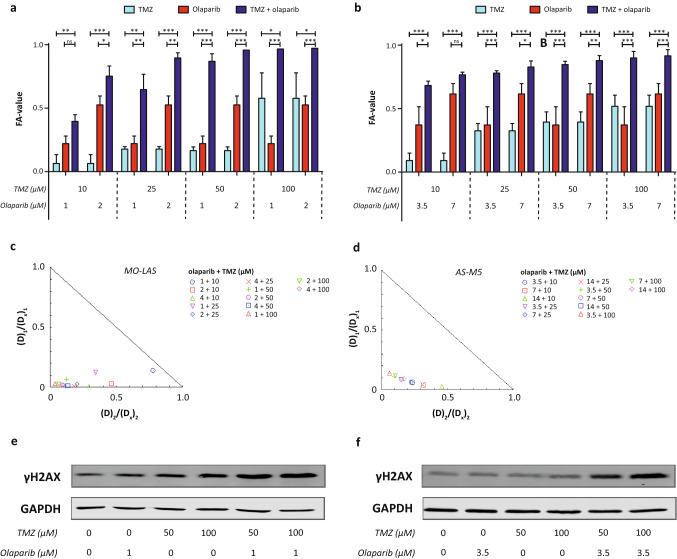
Combination therapy of olaparib and TMZ in MO-LAS and AS-M cell lines. **a** Cell viability of MO-LAS cells treated with single agent and combination treatment. **b** Cell viability of AS-M cells treated with single agent and combination treatment. **c** Isobologram of the combination treatment with increasing olaparib and TMZ concentrations in MO-LAS cells. The *X*- and *Y*-axis represent the fraction of the dose necessary as a single agent to generate reduction of *x*% cell viability (*D*_1/2_) divided by the portion of the drug in the combination treatment (*D*_1_ + *D*_2_) necessary to reduce a similar *x*% cell viability (*Dx*)_1/2_. *D*_1_ = TMZ, *D*_2_ = olaparib. **d** Isobologram of the combination treatment with increasing olaparib and TMZ concentrations in AS-M cells. **e** Protein expression of γH2AX in MO-LAS cells treated with single agent and combination treatment for 48 h. **f** Protein expression of γH2AX in AS-M cells treated with single agent and combination treatment for 48 h. *p value < 0.05; **p value < 0.01; ***p value < 0.001

MO-LAS cells showed an IC_50_-value of 1.99 ± 0.11 µM for olaparib and 110.3 ± 15.2 µM for TMZ (Table 2, Fig. 2a). AS-M cells exhibited a higher IC_50_-value for olaparib (6.62 ± 1.02 µM), and were more sensitive to TMZ than MO-LAS cells (IC_50_ 66.8 ± 11.9 µM) (Fig. 2b). The combination treatment significantly decreased cell viability compared to the single agent treatments in both cell lines (Fig. 3a, b), except for the combination of 1 µM olaparib and 10 µM TMZ in MO-LAS cells, and 7 µM olaparib and 10 µM TMZ in AS-M cells. Calculation of the combination index showed synergy (CI < 1.0) for all combinations in MO-LAS and AS-M cells (Fig. 3c, d). The combinations with 50 and 100 µM TMZ yielded even strong synergy (CI 0.1–0.3) in both cell lines except for 50 µM TMZ with 1 µM olaparib in MO-LAS (Table 3). For each combination, the DRI was > 1 for both olaparib and TMZ.

**Table 2:** IC_50_ values for olaparib and TMZ in MO-LAS and AS-M cell lines

Cell line	Compound	IC_50_ (mean ± SD)
MO-LAS	Olaparib (µM)	1.99 ± 0.11
	TMZ (µM)	110.3 ± 15.2
AS-M	Olaparib (µM)	6.62 ± 1.02
	TMZ (µM)	66.8 ± 11.9

*IC*_*50*_ concentration at which 50% of cell viability is affected, *SD* standard deviation, *TMZ* temozolomide

**Table 3:** FA, CI and DRI values for combination treatment in MO-LAS and AS-M cell lines

	Olaparib (µM)	TMZ (µM)	FA value (mean ± SD)	CI	DRI (TMZ; olaparib)
MO-LAS	1	10	0.396	0.918	(7.1; 1.3)
		25	0.752	0.494	(33.1; 2.2)
		50	0.647	0.470	(8.0; 2.9)
		100	0.896	0.230	(37.8; 4.9)
	2	10	0.869	0.194	(14.5; 8.0)
		25	0.957	0.117	(49.0; 10.4)
		50	0.966	0.072	(31.3; 25.2)
		100	0.972	0.094	(38.3; 14.8)
AS-M	3.5	10	0.682	0.291	(18.2; 4.2)
		25	0.766	0.359	(27.3; 3.1)
		50	0.780	0.235	(11.8; 6.7)
		100	0.828	0.290	(15.8; 4.4)
	7	10	0.847	0.211	(9.0; 10.0)
		25	0.878	0.244	(11.6; 6.4)
		50	0.899	0.206	(7.1; 15.4)
		100	0.916	0.224	(8.6; 9.3)

*FA value* fraction of cell viability affected by treatment, *SD* standard deviation, *CI* combination index, *DRI* dose reduction index, *TMZ* temozolomide

### Induction of DNA damage upon combination treatment

For further analysis of the effects of the combination treatment, we aimed to investigate those combination schedules with relatively low dosages of both compounds yet resulting in a large effect on cell viability, since lower dosages will be more likely to be suited for clinical application in the future. Based on these features, we selected the combination of 50% of the IC_50_ of olaparib with either 50 (low dose) or 100 µM (high dose) TMZ for both cell lines. Both cell lines were analyzed after 48 h of treatment, showing an increased level of DNA damage marker γH2AX after the combination treatment with both low and high dose TMZ, compared to the respective single agent treatments (Fig. 3e, f; Supplemental Fig. 3).

### Effects of combination treatment on apoptosis

**Fig. 4:**
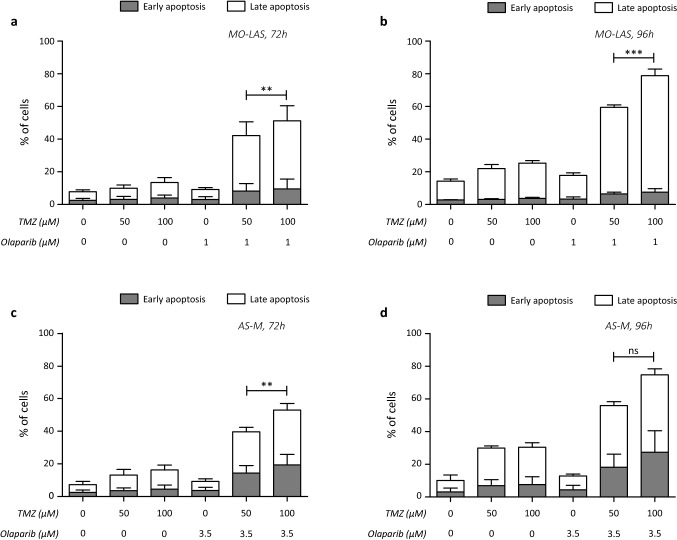
Apoptosis induction after combination therapy in MO-LAS and AS-M cell lines. **a** Induction of early and late apoptotic cells upon 72 h of treatment with low dose (50 µM) and high dose (100 µM) TMZ with olaparib in MO-LAS cells. **b** Induction of early and late apoptotic cells upon 96 h of treatment with low dose (50 µM) and high dose (100 µM) TMZ with olaparib in MO-LAS cells. **c** Induction of early and late apoptotic cells upon 72 h of treatment with low dose (50 µM) and high dose (100 µM) TMZ with olaparib in AS-M cells. **d** Induction of early and late apoptotic cells upon 96 h of treatment with low dose (50 µM) and high dose (100 µM) TMZ with olaparib in AS-M cells. *p value < 0.05; **p value < 0.01; ***p value < 0.001

Next, we aimed to study the effects of the combination schedules on apoptosis. After 72 h and 96 h of treatment, both combination with low and high dose TMZ significantly induced apoptosis compared to the single agent treatments in MO-LAS and AS-M cells (Fig. 4). The level of apoptosis was significantly higher after treatment with the high-dose TMZ combinations compared to the low-dose TMZ combinations, with exception of AS-M cells after 96 h, in which apoptosis was also increased but did not reach statistical significance.

## Discussion

In this study, we showed that PARP1 expression was present in almost all (46/47, 98%) UV AS samples and in both UV AS cell lines, indicating a potential role for PARP inhibition in UV AS. In addition, SLFN11 and PARP1 are present in a large proportion of non-UV AS. As expected, single agent olaparib yielded only modest effects in UV AS cell lines MO-LAS and AS-M. We therefore combined olaparib with the alkylating agent TMZ and demonstrated, synergy between olaparib and TMZ treatment in two UV AS cell lines, with strong synergy (CI < 0.3) in nearly all combinations using 50 and 100 µM TMZ. Combination treatment of olaparib and TMZ effectively induced apoptosis after 72 h and 96 h of treatment. The level of γH2AX, indicative of DNA damage, was significantly higher upon combination treatment compared to any of the single agents.

The DRI was > 1 for all combination treatments, indicating that the dosage of both TMZ and olaparib can be reduced in combination treatment compared to the effects of the respective single agent treatments. Given the potential toxicity of the combination treatment, these dose reductions could contribute to better tolerability while maintaining optimal anti-cancer effects. Both UV AS cell lines showed a higher DRI for TMZ compared to the DRI for olaparib, which is in line with previous finding showing that olaparib has a potentiating effect on TMZ (Curtin and Szabo 2013). Treatment with TMZ leads to methylation of DNA at three positions: the O6 and N7 position of guanine and the N3-position of adenine. These methylpurines are then excised, resulting in single strand DNA breaks (SSBs). Inactivation of PARP1 inhibits repair of SSBs, thus potentiating the effect of TMZ (Curtin and Szabo 2013).

The in vitro activity of PARPi and TMZ combination treatment in UV AS is in line with previous studies of this combination in other sarcomas (Ewing sarcoma [ES], chordoma and desmoplastic small round cell tumor [DSRCT]) (Cao et al. 2019; van Erp et al. 2020; Smith et al. 2015). Both in chordoma and DSRCT moderate to strong synergy was observed, as well as increased apoptosis (Cao et al. 2019; van Erp et al. 2020). These studies showed adequate translation of in vitro–in vivo effectivity, yielding significantly reduced tumor growth upon the combination treatment. Ideally, our in vitro findings in UV AS would be further examined in an in vivo study. However, no such model is yet available, and given the concordant results in chordoma and DSRCT, it could be considered to proceed to a clinical study directly.

To date, phase II clinical studies in different malignancies such as small cell lung cancer (SCLC), melanoma and glioblastoma multiforme showed that the combination of a PARPi with TMZ was well tolerated but yielded mostly only modest responses (Lu et al. 2018). The maximum reported median PFS was 3.6 months in melanoma patients, which did not differ from response to TMZ monotherapy (Middleton et al. 2015). In ES, no antitumor activity was observed in a phase I/II trial of PARPi talazoparib and TMZ in 15 patients (Schafer et al. 2020). Of note, these patients were already heavily pre-treated (median of three previous treatments). In addition, in vivo combination treatment with PARPi and TMZ in 10 ES models was only effective in 5/10 models (Smith et al. 2015). There was no clear correlation between PARP levels and responsiveness to treatment, and other biomarkers were not mentioned.

A high potential candidate biomarker for response to PARPi and chemotherapy combination treatment is Schlafen-11 (SLFN11), which was discovered to correlate with sensitivity to DNA damaging agents (Zoppoli et al. 2012; Barretina et al. 2012). In our study, SLFN11 expression was present in both UV AS cell lines and in 80% of UV AS tumor samples. Several preclinical studies provided evidence for the role of SLFN11 in cytotoxic effects of PARPi and chemotherapy in for instance Ewing sarcoma and lung cancer (Iwasaki et al. 2019; Lok et al. 2017; Tang et al. 2015). However, a recent study showed no correlation between SLFN11 expression and olaparib activity in breast cancer cell lines and PDX models (Winkler et al. 2021), stressing the need for cancer-specific experiments to examine SLFN11 as a biomarker. Thus far, the two reported clinical studies examining SLFN11 as a biomarker have not shown conclusive results. Although one study did report SLFN11 expression to be associated with a better response to TMZ/PARPi combination treatment in SCLC patients, a very low cut-off value for SLFN11 expression was used (any presence of SLFN11-positive cells) (Pietanza et al. 2018). In another trial investigating a PARPi and irinotecan with or without TMZ in solid malignancies, SLFN11 had a significant association with best response in all patients, but no association was found between the intensity of SLFN11 expression and best response (Federico et al. 2020). Hence, further studies are needed to determine the appropriate clinical application of SLFN11 as a biomarker in AS.

Due to the limited availability of evidence with regard to the appropriate threshold for SLFN11 positivity, we applied a cut-off value of 50% positive cells in the current study, aiming for a relevant level of expression. This might have resulted in an underestimation of SLFN11 positivity in UV AS cases. Although PARP1 and SLFN11 were not only present in UV AS cases but also in a large proportion of non-UV AS cases, further preclinical experiments in non-UV AS were not possible due to a lack of models.

Future research should further explore the use of SLFN11 as a biomarker in AS, starting with knockdown experiments in AS cell lines and establishment of the appropriate threshold for SLFN11 expression. Validation of SLFN11 in a clinical setting (either in AS or in other, more common cancers in which patient inclusion is less challenging) may enable the design of a biomarker-driven basket study to investigate PARPi and TMZ combination therapy in cancer patients with SLFN11-positive tumors. Ideally, such a study would only include cancer types in which in vitro results already indicate sensitivity to PARPi and TMZ treatment. Another potentially interesting development lies in the combination of PARPi with immune checkpoint inhibition (ICI), based on increased tumor mutational burden (TMB) and upregulation of Programmed Death-Ligand 1 upon PARP inhibition (Peyraud and Italiano 2020). Given the presence of PARP1 expression in our study, and the previously reported high TMB and corresponding clinical response to ICI in UV AS (Painter et al. 2020), this combination treatment could also be of interest for UV AS patients.

Overall, our data show that the combination therapy of PARPi olaparib with alkylating agent TMZ works synergistically in UV AS cell lines. In addition, we demonstrated expression of SLFN11, a biomarker for response to DNA damaging agents, in 74% of UV AS tumor samples. Therefore, these results may provide the first step towards novel AS subtype-specific targeted treatment options.

## Supplementary Information

Below is the link to the electronic supplementary material.

Supplementary file1 (DOCX 880 kb)

## Data Availability

Data and material are available upon request.
