# Supplementary material for: PARP inhibition in UV-associated angiosarcoma preclinical models
Source: J Cancer Res Clin Oncol. 2021 Jun 3;147(9):2579–90. doi: 10.1007/s00432-021-03678-4 (PMC8310857; doi:10.1007/s00432-021-03678-4)
Supplement: Supplementary file 1 — Supplementary file1 (DOCX 880 kb) [file 432_2021_3678_MOESM1_ESM.docx]

**Supplemental material**

Journal of Cancer Research and Clinical Oncology

Title: *PARP inhibition in UV associated angiosarcoma preclinical models.*

Authors: Marije E Weidema, Ingrid ME Desar, Melissa HS Hillebrandt-Roeffen, Anke EM van Erp, Mikio Masuzawa, PALGA-group, Uta E Flucke, Winette TA van der Graaf,, Yvonne MH Versleijen-Jonkers

Corresponding author: Marije Weidema, Department of Medical Oncology, Radboud University Medical Center, Nijmegen, The Netherlands, Marije.Weidema@radboudumc.nl

Supplemental Table 1. Clinical characteristics

| **Variable** | **UV AS patients (n=47)** | **Other AS patients (n=96)** |
| --- | --- | --- |
| Median age  (range, years) | 78 (50-98) | 71 (35-95) |
| Gender |  |  |
| Male | 28 (60%) | 32 (33%) |
| Female | 19 (40%) | 64 (67%) |
| Distant metastases |  |  |
| No | 31 (66%) | 60 (62%) |
| Yes | 8 (17%) | 12 (13%) |
| Unknown | 8 (17%) | 24 (25%) |
| Median overall survival (range, months) | 13 (0-194) | 11 (0-277) |
| AS subtype |  |  |
| UV AS | 47 (100%) | - |
| Cutaneous non-UV AS | - | 19 (18% |
| RT AS | - | 32 (33%) |
| Stewart Treves AS | - | 16 (17%) |
| Visceral AS | - | 29 (30%) |

Supplemental Table 2 Correlation of SLFN11 and PARP1 expression in all AS cases

|  | PARP1 + | PARP1 - | Not evaluable | Total |
| --- | --- | --- | --- | --- |
| SLFN11 + | 93 | 2 | 3 | 98 |
| SLFN11 - | 30 | 4 | 6 | 40 |
| Not evaluable | 3 | 1 | 1 | 5 |
| Total | 126 | 7 | 10 | 143 |

Supplemental figure 1. Distribution of SLFN11 expression

Supplemental figure 2. Western blot expression of PARP1 and SLFN11 in UV AS cell lines.


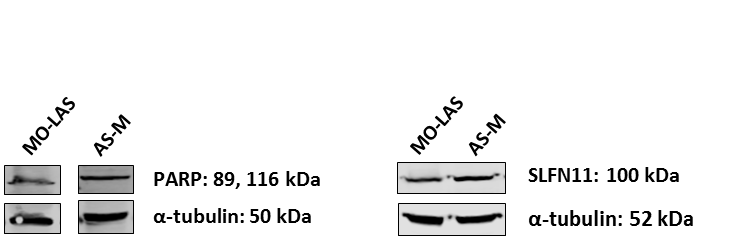


Left: Western blot showing PARP expression in MO-LAS and AS-M cells. Right: Western blot showing SLFN11 expression in MO-LAS and AS-M cells.

Supplemental figure 3. Quantification of γH2AX expression


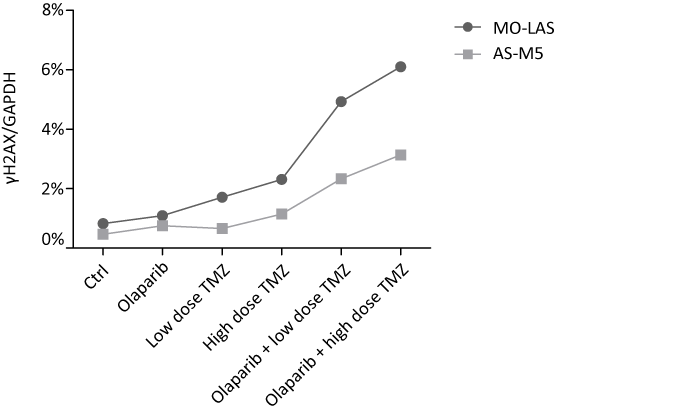


Quantification of γH2AX expression in MO-LAS and AS-M cells after single agent and combination treatment. γH2AX expression is depicted as a percentage of the loading control.
